# The effect of crocin supplementation on glycemic control, insulin resistance and active AMPK levels in patients with type 2 diabetes: a pilot study

**DOI:** 10.1186/s13098-020-00568-6

**Published:** 2020-07-09

**Authors:** Vahideh Behrouz, Ali Dastkhosh, Mehdi Hedayati, Meghdad Sedaghat, Maryam Sharafkhah, Golbon Sohrab

**Affiliations:** 1 Department of Clinical Nutrition and Dietetics, Faculty of Nutrition and Food Technology, National Nutrition and Food Technology Research Institute, Shahid Beheshti University of Medical Sciences, West Arghavan Street, Farahzadi Blvd., P.O. Box:19395-4741, Tehran, Iran; 2 Cellular and Molecular Endocrine Research Center, Research Institute for Endocrine Sciences, Shahid Beheshti University of Medical Sciences, Tehran, Iran; 3 Department of Internal Medicine, Imam-Hossein General Hospital, Shahid Beheshti University of Medical Sciences, Tehran, Iran; 4 Digestive Oncology Research Center, Digestive Diseases Research Institute, Shariati Hospital, Tehran University of Medical Sciences, Tehran, Iran

**Keywords:** Crocin, Diabete, Glycemic control, AMPK, Insulin resistance

## Abstract

**Background:**

Crocin as a carotenoid exerts anti-oxidant, anti-inflammatory, anti-cancer, neuroprotective and cardioprotective effects. Besides, the increasing prevalence of diabetes mellitus and its allied complications, and also patients' desire to use natural products for treating their diseases, led to the design of this study to evaluate the efficacy of crocin on glycemic control, insulin resistance and active adenosine monophosphate-activated protein kinase (AMPK) levels in patients with type-2 diabetes (T2D).

**Methods:**

In this clinical trial with a parallel-group design, 50 patients with T2D received either 15-mg crocin or placebo, twice daily, for 12 weeks. Anthropometric measurements, dietary intake, physical activity, blood pressure, glucose homeostasis parameters, active form of AMPK were assessed at the beginning and at the end of the study.

**Results:**

Compared with the placebo group, crocin improved fasting glucose level (*P* = 0.015), hemoglobin A1c (*P* = 0.045), plasma insulin level (*P* = 0.046), insulin resistance (*P* = 0.001), and insulin sensitivity (*P* = 0.001). Based on the within group analysis, crocin led to significant improvement in plasma levels of glucose, insulin, hemoglobin A1c, systolic blood pressure, insulin resistance and insulin sensitivity. The active form of AMPK did not change within and between groups after intervention.

**Conclusions:**

The findings indicate that crocin supplementation can improve glycemic control and insulin resistance in patients with T2D. Further studies are needed to confirm these findings.

*Trial Registration* This study has been registered at Clinicaltrial.gov with registration number NCT04163757

## Background

Diabetes has become a critical health issue throughout the world and one of the leading causes of disability, morbidity and mortality. Despite advances in knowledge and therapeutic approaches, significant challenges associated with management of diabetes remain [1, 2]. Increased incidence of diabetes mellitus along with insufficiency of the Conventional antidiabetic medications in management of disease have led to the search for alternative strategies for diabetes treatment [3]. Changes in lifestyle behaviors and dietary habits have immensely contributed to the prevention and treatment of diabetes mellitus. The effects of some dietary components on amelioration of diabetes have been shown previously [1, 4–7].

Crocin, a phytochemical component, is the main bioactive constituent of *Crocus sativus linne* (saffron) and *Gardenia jasminoides* [8]. It is a water soluble carotenoid compound and has the structure as an ester of disaccharide gentiobiose with dicarboxylic acid crocetin [9, 10]. Crocin possesses a wide range of pharmacological and therapeutic properties including anti-oxidant or radical scavenger, anti-inflammatory, anti-cancer, anti-depressant, neuroprotective and cardioprotective effects [11–16]. There are evidence demonstrating that crocin has protective effects against diabetes mellitus, hyperlipidemia, metabolic syndrome and obesity [17–22]. In animal model, crocin showed significant and dose-dependent antihyperglycemic and antioxidant activities in diabetic rat [23]. Shirali et al. reported that crocin improved insulin sensitivity and serum glycemic profile in animals with diabetes and modified lipid profile through enhancing insulin receptors sensitization [24]. Further, crocin has been shown to prevent elements of metabolic syndrome in rats via enhancing adenosine monophosphate-activated protein kinase (AMPK) [17]. Activation of AMPK by pharmacological or natural agents may reverse the metabolic abnormalities associated with diabetes mellitus [25, 26].

Current evidence supports the hypothesis that crocin can play an imperative role in the management of diabetes mellitus and allied complications such as oxidative stress and inflammation. Despite these promising results, to the best of our knowledge, no randomized clinical trial to date has been designed to show the effect of crocin supplementation on diabetes mellitus. This study evaluated the hypothesis that crocin supplementation would effectively improve metabolic abnormalities, and activate AMP-activated protein kinase when given orally to patients with diabetes mellitus.

## Methods and materials

### Study design and participants

This study was a randomized, double-blind, single-center, parallel-group, controlled clinical trial. The research protocol was approved by the Ethics Committee of the National Nutrition and Food Technology Research Institute at Shahid Beheshti University of Medical Sciences (IR.SBMU.nnftri.Rec.1398.009). This trial was registered at Clinicaltrial.gov under the identification number: NCT04163757 and has been carried out in accordance with The Code of Ethics of the World Medical Association (Declaration of Helsinki). Participants were recruited from the Imam Hossein Hospital in Tehran, Iran, from January to June 2019. The study protocol was explained for eligible subjects, and written informed consent was signed by all patients if they agreed to participate in the study. Participants could withdraw from the study by their own decision without any penalty.

This trial was performed in patients aged between 30 and 70 years, with a clinical diagnosis of diabetes mellitus (1–10 years) according to Standards of Medical Care in Diabetes Guidelines (American Diabetes Association) [27], body mass index (BMI) between 18.5 and 30 kg/m^2^, and also the use of oral hypoglycemic agents for controlling diabetes. Participants who took insulin, herbal and/or nutritional supplements, glucocorticoids, and non-steroid anti-inflammatory drugs within 3 months before the start of the study and during the study were excluded from the study. Other major criteria for exclusion were clinically diagnosed chronic diseases such as renal, hepatic, cardiovascular, autoimmune or any kind of inflammatory diseases, being pregnant or lactating, being on a weight loss diet within last 6 months, and having uncontrolled diabetes (HbA1c ≥ 8.5%).

### Randomization and treatment

**Fig. 1:**
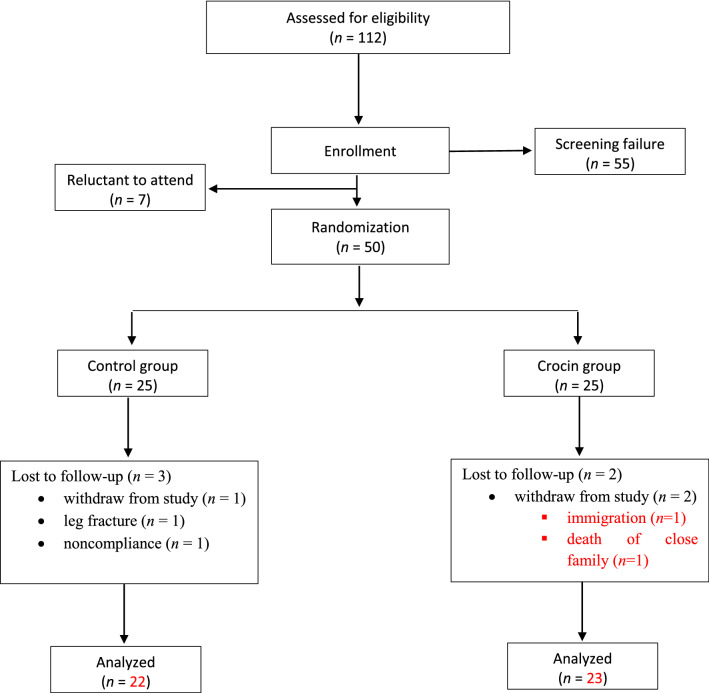
The study consort flowchart

A total of 112 patients were assessed for eligibility for inclusion in the trial. Fifty-seven of 112 subjects met the inclusion criteria, seven of whom were reluctant to attend (consort diagram, Fig. 1). Patients were selected using a simple sampling procedure and stratified (1:1) into two groups based on their sex and age, they were randomly allocated to receive either the crocin supplement (n = 25) or the placebo supplement (n = 25) for 3 months. Randomization sequence was computer-generated by a blinded biostatistician who was not involved with recruitment, using permuted block randomization (block size 4) and given to the interviewer. Patients were randomly assigned to the trial groups in accordance with the randomization list (code letters A or B) in chronological order. All investigators, staff related to the care of the patients, and participants were blinded to the treatment assignment until the final statistical analysis was completed.

The intervention group was administered orally 2 tablets of 15 mg crocin (Samisaz CO., Mashhad, Iran), and the control group was given two tablets of placebo (starch), with main meals (breakfast and dinner), for 12 weeks. Placebo tablets were similar to the crocin supplements in terms of the size, color, shape, smell and distribution bottles. Crocin was extracted from saffron stigmas using crystallization method with a purify more than 97%.

### Sample size

According to standard formula suggested for parallel clinical trials, the sample size was calculated for the glucose level, which was based on detection of 25 mg/dL difference in the mean glucose levels and standard deviation 29 mg/dL with a power of 80% (β = 20%) and a significance level of 0.05, yielding an estimated sample size of 23 for each group [28]. Due to the potential loss of samples, 25 patients were included in each treatment group.

### Follow-up assessments and compliance

Follow-up assessments were performed at every 4 weeks following initiation of the study. Anthropometric measurements, food records (3 days), physical activity assessment and tablets counts were assessed at each follow-up visit. Adherence to study treatment and adverse events were also ascertained at each visit. Compliance with consumption of tablets during the study was determined by counting the remaining tablets in each visit and weekly telephone call. Participants who had not consumed at least 90% of the expected tablets were regarded as noncompliance, which resulted in exclusion from the trial. The subjects were instructed to maintain their usual lifestyle and dietary habits during the study.

### Clinical, para-clinical and dietary intake assessment

At baseline, a general questionnaire was completed during a personal interview for every patient about demographics, medical history, medication use and health status. Height, body weight, waist and hip circumferences, and body fat percentage (BFP) were measured by an expert nutritionist at baseline, each follow-up visit, and the end of the study. Body weight and height were measured using a calibrated Seca balance measuring scale with stadiometer while participants wearing light clothes and no footwear. Waist and hip circumferences were measured using constant tension measuring tape and according to a standardized method. BFP was determined by Bioelectrical Impedance Analysis (BIA) method using a portable electrical micro-current monitor. Body mass index (BMI) and waist-to-hip ratio (WHR) were calculated according to WHO recommendation [29, 30].

Blood pressure measurements were also taken from each patient in a sitting position, twice with a 10-min interval, and the mean systolic and diastolic blood pressure measurements were used for analysis. Dietary intake of each patient was collected using 3 day 24 h dietary record (2 weekdays and 1 weekend) in months 0 and 3 [31]. These records were verified by a dietitian, and then analyzed by Nutritionist IV (First Databank, Hearst Corp, San Bruno, CA, USA). Physical activity was also evaluated by using a validated semi-quantitative questionnaire, based on metabolic equivalent (MET)-min/day values [32].

Biochemical testing was performed on each patient at the beginning and end of the study, after 12 h overnight fasting. All blood samples divided 2 parts, 1 mL for HbA1c determination and the second remained parts were immediately centrifuged (3500 rpm:10 min at room temperature) and the separated sera was stored at − 80 ℃ for subsequent biochemical analysis. All biochemical parameters were assessed in same laboratory by using standard laboratory methods. Fasting glucose concentration was measured by using GOD/POD method. Fasting insulin concentrations were determined using the enzyme-linked immunosorbent assay (ELISA) (Demeditec Diagnostics, Germany) with a lower sensitivity limit of 1.76 µIU/mL. The hemoglobin A1c (HbA1c) was measured on the whole blood sample by direct enzymatic HbA1c assay (Diazyme Laboratories, Inc., CA, USA). HOMA-IR, QUICKI, and HOMA-β were calculated to estimate insulin resistance, insulin sensitivity, and β-cell homeostasis, respectively [33, 34]. Phosphorylated AMP-activated protein kinase (P-AMPK) was measured in peripheral blood mononuclear cell by using an ELISA kit (ZellBio, Ulm, Germany) according to the manufacturer’s protocol.

### Statistical analysis

In this study, statistical analysis of data was performed by SPSS software version 24. Normality of data distribution was checked through the Kolmogorov–Smirnov test. Results of categorical variables were presented as frequency, and continuous data were shown as mean ± SD. Student’s *t* test was done to detect differences between groups. For within-group comparison, paired *t* test was used. Parameters with skewed distribution were natural logarithm-transformed (ln) to normalize distribution.

To remove the effects of confounding factors, analysis of covariance (ANCOVA) was used to determine any differences at the end of the study with adjusting for baseline values and height. Statistical significance was defined at *P* < 0.05, based on two-sided tests.

## Results

From fifty eligible patients, 45 (90%) completed the 3 months study period. Among the patients in the crocin group, 2 patients were excluded (withdraw from study due to immigration, n = 1, and death of close family member, n = 1). Three individuals in the placebo group were excluded (withdraw from study, *n* = 1, leg fracture, n = 1, noncompliance, *n* = 1) (Fig. 1). There was no significant difference between the rates of dropout between the 2 groups. None of the participants reported any serious adverse events during intervention. As shown in Table 1, the baseline demographic and clinical data of both groups were similar except the height, which was significantly greater in the crocin group in comparison with placebo group (158.97 ± 6.93 vs. 155.11 ± 5.11, *P* = 0.04).

**Table 1:** Baseline characteristics at enrollment

Variable	Crocin group (n = 25)	Placebo group (n = 25)	*P* value*
Age (years)	57.08 ± 7.41	59.86 ± 9.46	0.122
Sex (M/F) (n)	4/21	3/22	> 0.999
Metabolic characteristics			
Height (cm)	158.97 ± 6.93	155.11 ± 5.11	0.04
Weight (kg)	77.08 ± 10.18	74.18 ± 7.97	0.296
Body fat (%)	41.18 ± 9.07	43.56 ± 7.61	0.329
BMI (kg/m^2^)	30.64 ± 4.79	30.85 ± 3.19	0.865
WC (cm)	104.65 ± 9.72	104.9 ± 8.84	0.927
WHR	0.96 ± 0.06	0.96 ± 0.05	0.813
Medication use [n (%)]			
Glucose-lowering medications			
Metformin	22 (88)	19 (76)	0.346
As single OHA	6 (26.1)	9 (40.9)	0.353
Sulfonylureas	12 (48)	7 (28)	0.231
As single OHA	0 (0)	1 (4.5)	0.489
Thiazolidinediones	0 (0)	2 (8)	0.233
Meglitinides	4 (16)	0 (0)	0.109
DPP-4 inhibitors	4 (16)	5 (20)	0.722
α-Glucosidase inhibitors	1 (4)	2 (8)	0.608
Lipid-lowering medications	15 (60)	17 (68)	0.514
Anti-hypertensive medications	10 (40)	11 (44)	0.768
Dietary factors			
Total energy (kcal)	1853.49 ± 483.73	1899.09 ± 467.18	0.749
Dietary carbohydrate (g/day)	242.08 ± 64.07	255.62 ± 79.22	0.531
Dietary protein (g/day)	61.48 ± 18.78	61.34 ± 21.86	0.982
Dietary fat (g/day)	73.75 ± 33.28	74.34 ± 23.35	0.945
Dietary cholesterol (mg/day)	184.42 ± 98.87	203.01 ± 112.23	0.558
Dietary fiber (g/day)	16.22 ± 5.36	17.94 ± 5.07	0.277
Vitamin A (mg/day)	567.06 ± 356.17	821.43 ± 715.09	0.247
Β-carotenoid (mg/day)	285.05 ± 265.38	401.34 ± 666.84	0.982
MET (min/day)	741.58 ± 395.72	874.4 ± 857.06	0.725
Systolic blood pressure (mmHg)	133.86 ± 12.83	128.27 ± 18.49	0.248
Diastolic blood pressure (mmHg)	79.92 ± 8.22	78.95 ± 14.64	0.346

*BMI* body mass index, *WC* waist circumference, *WHR* waist to hip ratio, *MET* metabolic equivalent of tasks, *DPP-4* dipeptidyl peptidase 4, *OHA* oral hypoglycemic agent

Mean ± SD (all such values)

^*^ Based on independent t test for continuous data and Chi square test for categorical variables, both groups were well matched in respect to baseline characteristics, except for height

**Fig. 2:**
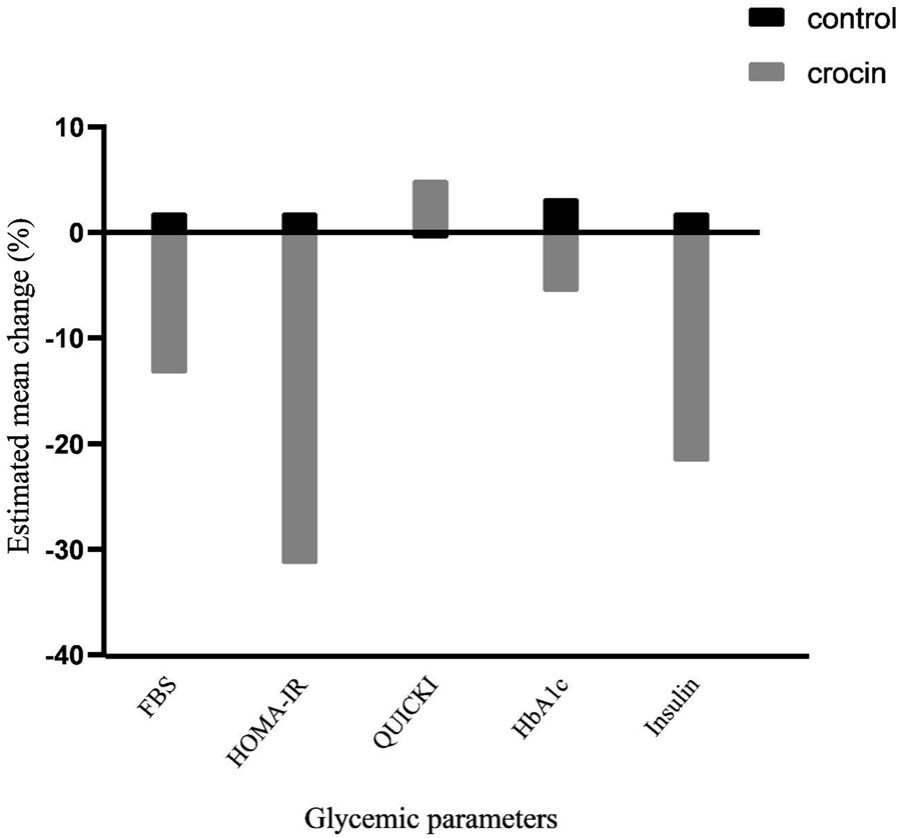
Estimated mean percent change of glycemic parameters

The estimated mean changes in glycemic parameters and insulin resistance of two groups are depicted in Fig. 2. As presented in Table 2, systolic and diastolic blood pressure showed no significant changes between the two groups during the study. However, a significant improvement in SBP was seen in the crocin group in comparison with baseline values (*P* = 0.002).

**Table 2:** changes in clinical and paraclinical tests

Variables	Group	Before	After	Mean changes (95% CIs)	*P* value^a^	*P* value^b^
SBP (mmHg)	Crocin	133.86 ± 12.83	123.06 ± 17.12	− 10.8 (− 16.79, − 4.58)	0.002	0.092
	Control	128.27 ± 18.49	126.06 ± 19.64	− 2.2 (− 6.21, 1.47)	0.298	
DBP (mmHg)	Crocin	79.92 ± 8.22	77.2 1± 10.55	− 2.7 (− 6.59, 1.52)	0.149	0.750
	Control	78.95 ± 14.64	77.25 ± 12.44	− 1.7 (− 6.97, 3.19)	0.758	
FBS (mg/dL)	Crocin	148.73 ± 30.07	129.26 ± 29.31	− 19.47 (− 30.23, − 8.32)	0.002	0.015
	Control	157.18 ± 63.29	160.18 ± 57.34	3 (− 22.19, 23.55)	0.249	
Insulin (mU/L)	Crocin	17.27 ± 7.14	13.51 ± 4.62	− 3.75 (− 6.16, − 1.33)	0.021	0.046
	Control	14.95 ± 5.52	15.2 5± 5.04	0.29 (− 1.28, 2.29)	0.931	
QUICKI	Crocin	0.12 ± 0.008	0.13 ± 0.007	0.006 (0.003, 0.009)	0.001	0.001
	Control	0.13 ± 0.007	0.13 ± 0.007	− 0.0009 (− 0.003, 0.001)	0.433	
HOMA−IR	Crocin	6.33 ± 2.85	4.34 ± 1.95	− 1.99 (−2.95, −1.07)	0.001	0.001
	Control	5.74 ± 2.85	5.86 ± 2.43	0.11 (− 0.64, 0.85)	0.858	
HOMA−β	Crocin	81.75 ± 42.49	92.99 ± 70.92	11.24 (− 13.98, 39)	0.330	0.930
	Control	81.67 ± 60.54	88.45 ± 91.08	6.77 (− 12.07, 27.63)	0.910	
HbA1c	Crocin	7.80 ± 1..29	7.36 ± 1.47	− 0.44 (− 0.90, − 0.02)	0.071	0.045
	Control	7.61 ± 1.62	7.86 ± 1.75	0.25 (− 0.22, 0.71)	0.303	
pAMPK (pg/mg protein)	Crocin	34.55 ± 53.52	18.20 ± 7.69	− 16.34 (− 40.64, 0.56)	0.378	0.433
	Control	27.72 ± 20.57	20.14 ± 10.81	− 7.57 (− 18.5, 1.74)	0.131	

*SBP* systolic blood pressure, *DBP* diastolic blood pressure, *FBS* fasting blood sugar, *QUICKI* quantitative insulin sensitivity check index, *HOMA-IR* homeostasis model assessment for insulin resistance, *HOMA-β* homeostasis model assessment of β-cell function, *HbA1c* hemoglobin A1c, *pAMPK* phospho-adenosine monophosphate-activated protein kinase

^a^Based on paired *t* test for within group comparison

^b^Based on ANCOVA model that regressed changes from baseline on treatment group, baseline value of the outcome and height

Compared with the placebo group, participants taking crocin tablets had a significantly greater decrease in the following glycemic parameters: FBS (148.73 ± 30.07 to 129.26 ± 29.31 in the crocin group and 157.18 ± 63.29 to 160.18 ± 57.34 in the placebo group; *P* = 0.015), insulin (17.27 ± 7.14 to 13.51 ± 4.62 in the crocin group and 14.95 ± 5.52 to 15.25 ± 5.04 in the placebo group; *P* = 0.046), HOMA-IR (6.33 ± 2.85 to 4.34 ± 1.95 in the crocin group and 5.74 ± 2.85 to 5.86 ± 2.43 in the placebo group; *P* = 0.001), whereas no significant difference was found within and between the two groups in HOMA-β (*P* = 0.930). Moreover, insulin sensitivity (QUICKI) increased significantly in the crocin group compared with those in the baseline (*P* = 0.001) and the placebo group (*P* = 0.001) at the end of the study. As expected, crocin supplementation resulted in significantly greater improvement in glycated hemoglobin as compared to the placebo group (*P* = 0.045). pAMPK did not show any significant changes within and between groups during study.

## Discussion

To the best of our knowledge, this trial is the first randomized, double-blind, placebo-controlled clinical trial designed to investigate the possible efficacy of crocin supplementation in management of diabetes mellitus and addressed some of its mechanisms of action. Our findings indicate that crocin could improve glycemic parameters and insulin resistance in patients with type-2 diabetes, after 3 month consumption of crocin supplements. Crocin supplementation significantly reduced systolic blood pressure at the end of intervention compared with baseline values, however our study showed no significant changes in levels of pAMPK between groups at the of the study.

Animal studies have shown that crocin can ameliorate the plasma glycemic profile such as improving insulin sensitivity, augmenting insulin secretion, and reducing glycosylated hemoglobin [24, 35–37]. The ability of crocin in altering the phosphorylation of acetyl-CoA carboxylase (AMPK/ACC) and mitogen-activated protein kinases justifies these effects to some extent [16]. Similarly, crocin can improve the metabolism of glucose and thereby result in glycemic control through downregulation of tumor necrosis factor-ɑ (TNF-ɑ). An increase in leptin mRNA and protein has also been reported following the crocin supplementation in animal models of diabetes mellitus [37]. On the other hand, the protective effect of crocin on glycemic control can be attributed to induction of the GLUT4 expression/localization, improvement in β-cell function, lowering of free fatty acids and triglycerides, and suppression of inflammatory responses [36, 38–41]. In support of our finding, in an experiment conducted by Shirali et al. [24], crocin could significantly decrease hemoglobin A1c and insulin resistance in diabetic rats, probably through prevention of oxidative stress and improvement of lipid profile. These results are in agreement with the work of Sepahi et al. [42], which found daily administration of crocin (15 mg) markedly decreased hemoglobin A1c in patients with diabetes mellitus. In contrast, supplementation with 100 mg crocin for 6 weeks in patients with metabolic syndrome had no effect on the components of metabolic syndrome [43]. It may be due to the short duration of intervention, differences in the designs of studies, confounding factors, or dissimilar examined populations.

AMPK is a central regulator of energy metabolism and nutrient status that regulates glucose and lipid metabolism. Downregulation of AMPK is associated with obesity, metabolic syndrome and diabetes mellitus [44]. In our study, oral administration of crocin could not alter the active form of AMPK enzyme compared to placebo, moreover no significant changes were observed within each group. Experimental studies have exposed promising results regarding the effect of crocin on pAMPK. Algandaby [17] has shown the protective effects of crocin against metabolic syndrome through activation of AMPK and peroxisome proliferator-activated receptor-γ. Administration of 10 mg/kg body weight of crocin restored the active form of AMPK level in comparison with control group. This report lend support to Luo et al. study [8]. Despite the promising findings of animal experimental models [8, 17], our intervention failed to make a significant activation of AMPK. To the best of our knowledge, no clinical trial to date has been conducted on the effects of crocin on overexpression of AMPK in patients with diabetes mellitus. The difference in findings may be due to the low dosage of crocin or small number of participants in our research.

Moreover, we observed systolic and diastolic BP were unchanged and not influenced by crocin administration at the end of intervention period. However, there was a significant reduction in SBP in those taking crocin at the end of intervention compared with baseline values. The findings of previous clinical trials [21, 45] are in line with the present study. In vitro studies indicated that administration of crocin, saffron or safranal could reduce blood pressure in a dose-dependent manner [46–48]. Antihypertensive effects of saffron and its main constituents are partly due to blocking of calcium channels, interaction with endothelial nitric oxide, and antioxidant activity [49]. These discrepancies in human and animal studies may be explained by different conditions existing in each study.

The current clinical trial has some strengths including a high compliance of the participants (> 90%), double-bind, placebo-controlled design, equal sex and age distribution, a moderate to low dropout rate, and assessment of active form of AMPK in peripheral blood mononuclear cells. Moreover, according to our knowledge, this study is the first clinical trial that investigates the effects of crocin in patients with type-2 diabetes. However, this trial had some limitations. Due to the short duration of follow-up, it is not possible to be certain that these positive alterations in glycemic parameters would be sustained. Although the number of participants in each group was adequate for statistical analysis in this trial, but larger study groups would be desirable.

## Conclusions

In conclusion, this randomized, double-blind, placebo-controlled, clinical trial indicated some evidence that 30 mg/day crocin supplementation could improve glucose homeostasis and insulin resistance for treatment of T2D, and it can be recommended as an adjuvant to standard diabetes care. Although the findings of this trial confirmed our assumptions, further clinical studies are required to find the efficacy, ideal dose and duration of crocin supplementation in patients with T2D, and to investigate possible mechanisms.

## Data Availability

The datasets used during the current study are available from the corresponding author on reasonable request.

## Abbreviations

BMI: Body mass index
WC: Waist circumference
WHR: Waist to hip ratio
MET: Metabolic equivalent of tasks
SBP: Systolic blood pressure
DBP: Diastolic blood pressure
FBS: Fasting blood sugar
QUICKI: Quantitative insulin sensitivity check index
HOMA-IR: Homeostasis model assessment for insulin resistance
HOMA-β: Homeostasis model assessment of β-cell function
HbA1c: Hemoglobin A1c
pAMPK: Phospho-adenosine monophosphate-activated protein kinase
